# Disease of the Antrum

**Published:** 1870-04

**Authors:** 


### ARTICLE IV.

### *Disease of the Antrum.*

J. H. M., of Surry Co., North Carolina, sent us, a few weeks ago, the following history of his own case:

"In 1859. I experienced severe toothache in the left superior 1st bicuspid, followed by swelling. In about a month suppuration occurred, and the pus was discharged through the left nostril, and has continued to run, with short intermission until the present time.

I have no acute pain, but there appears to be a fullness and a dull aching on the side of the left nostril, which appears to be the seat of the disease, most of the time. In the morning the matter appears to run into my throat and mouth, and smells very offensive; in fact, this is the case all the time.

My general health is not good; I am very nervous, with constant weakness in the back; and although my appetite is good, I am considerably emaciated, yet able to do light work in good weather.

In 1864 a Dentist extracted all the teeth on the affected side back of the eye-tooth, and punctured the antrum from the socket of the first molar without any beneficial effect. The soreness is mostly above the eye tooth, which tooth has always been somewhat sore, so much so as to lead me to suspect it is diseased about the end of the root.

I have been treated by physicians with iodine, &c., without effect. They say I have neuralgia also, but think it originates mostly from the antrum."

We advised, in the first place, the removal of the affected cuspid tooth, believing it necessary to get rid of all irritants, and to determine, by probing, whether an opening existed from the cavity of this tooth into the antrum. If no such communication was discovered, then to perforate the bone above the point formerly occupied by the palatine root of the first molar on the affected side, and use as injections, either Lugol's solution, or the permanganate of potash; to inhale iodine once or twice a day, and paint the affected side of nostril and part of face with tincture of iodine; internally to use iodide of iron, iodide of potass, and cod liver oil, taking this prescription three times a day; also to use the bitter tonic tincture of gentian half an hour before each meal.

